# Chromatin Regulation and Gene Centrality Are Essential for Controlling Fitness Pleiotropy in Yeast

**DOI:** 10.1371/journal.pone.0008086

**Published:** 2009-11-30

**Authors:** Linqi Zhou, Xiaotu Ma, Michelle N. Arbeitman, Fengzhu Sun

**Affiliations:** 1 Department of Biological Sciences, University of Southern California, Los Angeles, California, United States of America; 2 Department of Molecular and Cell Biology, University of Texas at Dallas, Richardson, Texas, United States of America; 3 MOE Key Laboratory of Bioinformatics and Bioinformatics Division, TNLIST/Department of Automation, Tsinghua University, Beijing, People's Republic of China; University of Glasgow, United Kingdom

## Abstract

**Background:**

There are a wide range of phenotypes that are due to loss-of-function or null mutations. Previously, the functions of gene products that distinguish essential from nonessential genes were characterized. However, the functions of products of non-essential genes that contribute to fitness remain minimally understood.

**Principal Findings:**

Using data from *Saccharomyces cerevisiae*, we investigated several gene characteristics, which we are able to measure, that are significantly associated with a gene's fitness pleiotropy. Fitness pleiotropy is a measurement of the gene's importance to fitness. These characteristics include: 1) whether the gene's product functions in chromatin regulation, 2) whether the regulation of the gene is influenced by chromatin state, measured by chromatin regulation effect (CRE), 3) whether the gene's product functions as a transcription factor (TF) and the number of genes a TF regulates, 4) whether the gene contains TATA-box, and 5) whether the gene's product is central in a protein interaction network. Partial correlation analysis was used to study how these characteristics interact to influence fitness pleiotropy. We show that all five characteristics that were measured are statistically significantly associated with fitness pleiotropy. However, fitness pleiotropy is not associated with the presence of TATA-box when CRE is controlled. In particular, two characteristics: 1) whether the regulation of a gene is more likely to be influenced by chromatin state, and 2) whether the gene product is central in a protein interaction network measured by the number of protein interactions were found to play the most important roles affecting a gene's fitness pleiotropy.

**Conclusions:**

These findings highlight the significance of both epigenetic gene regulation and protein interaction networks in influencing the fitness pleiotropy.

## Introduction

Mutations in individual genes or in a combination of genes can have varying effects on phenotype. To study this further, individual *S. cerevisiae* strains, each with a gene-deletion mutation for a gene in the genome, such that there is a strain with a mutation for every gene in the genome, have been generated [1]. The studies of the effects of these mutations on viability, when each strain was grown in rich medium, have identified a set of essential genes, consisting of about 20% of all the genes [1]. Essential genes are required for cell viability, while the other genes are nonessential genes. The essential genes have been found to encode products that have a large number of physical interaction partners [2], although this finding has been challenged [3]–[6], and are conserved across phyla [7]. The observation that ∼80% of genes are not essential for viability suggested that they contribute to optimum fitness in response to different growth conditions.

To study the functions of non-essential genes, growth rates (fitness) of the *S. cerevisiae* deletion strains have been examined in various culture conditions [8]–[10]. One of the objectives of these studies has been to group genes with similar fitness profiles, to provide insight into gene function. With these data sets, a gene's importance to survival can be measured by fitness pleiotropy. A gene's fitness pleiotropy is defined as the number of conditions that the fitness of the corresponding *S. cerevisiae* deletion strain is significantly reduced [11]. Fitness pleiotropy is a quantitative measurement of the importance of a gene's function to the organism's relative fitness. The more important a gene is to fitness, the higher the fitness pleiotropy. Thus, if the gene is important for growth, the gene should have a high fitness pleiotropy measure. Previously it has been shown that the fitness pleiotropy of a gene is positively associated with the number of biological processes that the gene's product functions in, as well as the number of protein interaction partners of the gene product [11], [12]. A positive association between fitness pleiotropy of transcription factors (TF) and the number of the TF's target genes has also been found [11]. However, the positive association was not statistically significant (p-value  = 0.22).

Here, the fitness data from the *S. cerevisiae* deletion strains from the previous studies [8]–[10] were re-examined to determine the effect of chromatin regulation on fitness pleiotropy in two ways. Chromatin regulation plays an important role in a gene's response to internal and external stimuli. First, we examined the fitness pleiotropy of genes that encode chromatin regulatory factors, that likely influence transcription by altering chromatin structure. Second, we examine the epigenetic regulatory effect for every gene, here defined as the chromatin regulation effect; CRE of a gene is a measure of the mean absolute change of the gene's expression level when chromatin regulators are mutated, as was done previously [13]. We find that CRE is strongly associated with fitness pleiotropy.

Genes that are important for fitness tend to have stable expression levels under many perturbations and thus it is expected and shown here that fitness pleiotropy is negatively associated with gene expression variation. Since the presence/absence of a TATA-box has been found to be the major contributor to expression variation [14]–[16], we also studied the relationship between fitness pleiotropy and the presence/absence of TATA-box, and show that they are highly associated. Additionally, we further examined the relationship between the number of target genes for TFs and their fitness pleiotropy, and showed that they are highly statistically significantly associated.

We also determined if other centrality measures, in addition to protein physical interaction (PPI) degrees, are associated with fitness pleiotropy. We considered two additional centrality measures: 1) betweenness (BW; defined as the fraction of shortest paths between any two proteins that pass through the given protein in a protein interaction network [17]) and 2) the clustering coefficient (CC; defined as the ratio of the number of edges between its first order neighbors, over all possible edges between its first order neighbors of a given protein [18]). Proteins in complexes tend to have high CC than other proteins. It has previously been shown that proteins within complexes are more likely to be essential [3]. Thus we consider three measures, PPI degree, BW and CC, whereas the previous studies have only considered one measure (PPI degree [11], [12]). Our results show that both PPI degree and CC are strongly associated with fitness pleiotropy and that the association between BW and pleiotropy can be explained by the association between PPI degree and pleiotropy.

In summary, the following work will demonstrate that 1) chromatin regulation, as measured by chromatin regulation effect (CRE), and 2) gene centrality, particularly in relation to the protein interaction network, as measured by PPI degree, are important contributors to fitness pleiotropy in *S. cerevisiae*.

## Results and Discussion

Three phenotypic profiles were used to define fitness pleiotropy. In the first experiment, a quantitative profile for 4,277 mutant diploid strains, each homozygous for a deletion of a nonessential gene, were examined under 51 growth conditions [8]. In the second experiment, a quantitative profile of 4,111 mutant haploid strains, each with a deletion of a nonessential gene, were examined under 82 growth conditions [9]. In the third experiment, a quantitative profile for 4,742 mutant strains each homozygous mutant for a deleted nonessential genes were examined under 418 conditions and a quantitative profile for 4,956 mutant strains each heterozygous for a deletion of a nonessential genes were examined under 726 conditions [10]. The results using the phenotypic profile from Brown et al. [8] are presented below, while those based on the phenotypic profiles from Parsons et al. [9] and from Hillenmeyer et al. [10] are found in the Files S2 and S3. The results based on phenotypic profiles of heterozygous deletions [10] are not shown since the statistical significance is weak or not observed in some relationships. Moreover, we found that the correlation of fitness pleiotropy for homozygous deletions [10] and heterozygous deletions [10] under 119 unique conditions was very low. The biological explanation for the differences observed in the heterozygous mutants compared to homozygous mutants is likely that most of these genes are haplosufficient under the growth conditions examined.

To ensure that our results do not depend on the particular interaction datasets used, we studied three interaction data sources: MIPS [19], DIP [20], and BioGrid [21]. In the main text, we only present the results with regard to protein interactions using the MIPS data set [19]; the results using DIP [20] and BioGrid [21] data sets are found in Files S1, S2 and S3.

### The Influence of Transcription Factors, Chromatin Regulators, and Chromatin Regulation Effect on Fitness Pleiotropy

Phenotypic changes are associated with changes in gene expression levels. Hence, genes with products that influence gene expression might also be associated with fitness pleiotropy, such as genes that encode transcription factors (TFs) or chromatin regulators (CR) that underlie epigenetic gene regulation. Epigenetic gene regulation refers to modification of chromatin by CRs, such as methylation or acetylation of histone proteins, a component of chromatin. Given that chromatin modification usually affects TF binding and thus gene expression regulation, it is hypothesized that both TFs and CRs must be important contributors to fitness pleiotropy. To compare the contributions of TFs or CRs to fitness pleiotropy, the influence of both gene and chromatin regulatory networks on fitness pleiotropy were examined.

**Figure 1:**
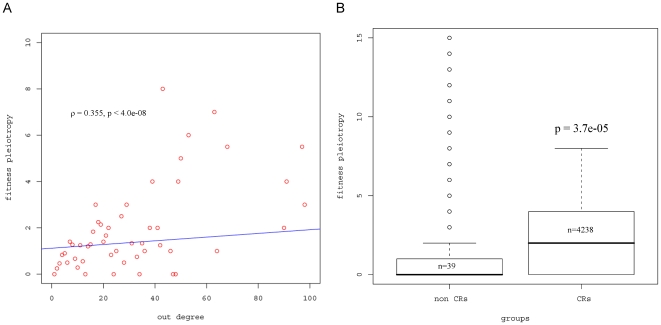
The relationship between fitness pleiotropy and different measurements. A) Fitness pleiotropy is positively associated with the number of targeted genes that each TF regulates (ρ = 0.355, p = 4.0e−08). Note that only less than 0.5% protein has out-degree higher than 100 (data not shown). B) Fitness pleiotropy for CRs and non CRs. The line in the box indicates the median value. The upper edge of the box indicates the 75^th^ percentile, and the lower edge indicates the 25^th^ percentile. The ends of the vertical line indicate the minimum and the maximum values, and the points outside the ends of the vertical line are outliers. P-values are given to test the hypothesis that the median fitness pleiotropy for CRs is higher than that for non CRs using non-parametric Wilcoxon rank sum test. The value of n in the box is the number of genes for each group.

First, transcription factors in gene regulatory networks were examined, in which the nodes are the genes, and directed edges indicate regulatory relationship. We used the gene regulatory network constructed in [22]. In such a network, there are two types of degrees, in-degree and out-degree. The in-degree of a gene measures the number of TFs that regulate the gene. The out-degree of a TF measures the number of genes that the TF regulates. When a TF is deleted, the genes regulated by the TF will be affected. Thus, if the out-degree of a TF is high, many genes will be affected when the TF is deleted, and consequently should increase fitness pleiotropy. Therefore, we expect that the fitness pleiotropy should increase with out-degree, but not in-degree. As shown in Figure 1A, fitness pleiotropy is significantly positively associated with the out-degree of TFs (ρ = 0.355, p = 4.0e−08). On the other hand, there is no significant association between fitness pleiotropy and in-degree in the gene regulatory network. This is expected as in-degree only indicates how many TFs control the gene, and it is not related to its effect on other genes and thus overall fitness. This result supports the observation that fitness pleiotropy was positively associated with the out-degree of TFs although the association was not significant in [11].

We next investigated the CRs that underlie chromatin modification, such as histone acetylation/methylation, ubiquitylation/deubiquitylation and phosphorylation. Given that chromatin modification has a high degree of impact on gene expression, it is expected that CR genes should have high fitness pleiotropy. To test this, 65 genes that encode chromatin regulators were identified from a previous study [23], and the median fitness pleiotropy of CR genes was found to be 2.282. This is significantly higher than the median fitness pleiotropy of non-CR genes (1.149) (p = 3.7e−5, Figure 1B). These results demonstrate the importance of genes that encode chromatin regulators relative to other genes, with respect to the organism's fitness.

We next studied the relationship between the potential for a gene to be chromatin regulated with the gene's pleiotropy. We used the following approach to measure the potential for a gene to be chromatin regulated. Here, we used the gene expression compendium that examined global gene expression profiles in 116 different *S. cerevisiae* strains that have CR genes mutated [23]. The potential for a gene to be CR-regulated was determined by the chromatin regulation effect (CRE) measure, which is defined as the mean absolute value of the logarithm of the gene expression changes across the 116 perturbations, as was previously done [16]. The CRE measures the likelihood of a gene to be epigenetically regulated. This means that, as CRE increases, the likelihood that this gene is epigenetically regulated also increases. It has been shown that CRE is significantly positively associated with gene expression variation, due to trans-regulation [16].

**Figure 2:**
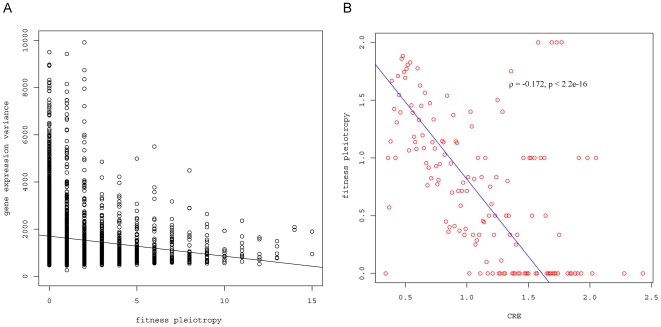
The relationship between fitness pleiotropy and different measurements. A) Fitness pleiotropy is significantly negatively associated with gene expression variation (ρ = −0.151, p<2.2e−16). B) Fitness pleiotropy is negatively associated with chromatin regulatory effect (CRE) (ρ = −0.172, p<2.2e−16). The red dots are the mean fitness pleiotropy of the genes, given CRE. For visualization, the blue line represents linear regression.

Here, fitness pleiotropy is negatively associated with gene expression variation suggesting that genes that show high expression variation across the experiments are less important for fitness (see Figure 2A). Therefore, we hypothesize that CRE will also be negatively associated with fitness pleiotropy. Based on the data, we studied the relationship between a gene's CRE and fitness pleiotropy and found that they are indeed significantly negatively associated (ρ = −0.172, p<2.2e−16, Figure 2B). Thus, genes that display high expression change when chromatin regulators are mutated tend to have low fitness pleiotropy. This result suggests that genes with high CRE might function under specific conditions. As a result, the deletion of such genes would result in defective growth only under specific conditions, and will have low fitness pleiotropy. The dataset was further examined to identify genes with low fitness pleiotropy that are also chromatin modified, to determine if this hypothesis is correct. Indeed, *pho5* (fitness pleiotropy  = 0) encodes acid phosphatase in budding yeast and is induced under phosphate starvation, but repressed under high-phosphate condition. It was found that the promoter of *pho*5 is protected by four positioned nucleosomes under high-phosphate conditions [24] and *pho5* activation is epigenetically regulated at intermediate phosphate concentrations [25]. Another example is *SSA3* (fitness pleiotropy  = 0), which encodes a member of the heat shock protein 70 (HSP70) family. The expression of *ssa3* is induced after diauxic shift or upon heat shock [24]. Previous studies have shown that there is a significant increase in H4 acetylation at the promoter of *ssa3* upon heat shock [26]. These two examples are consistent with the idea that genes that are epigenetically regulated and have products that function under specific conditions show low fitness pleiotropy.

Given that TF out-degree is positively associated with fitness pleiotropy (see above), the relative contributions of out-degree and CRE to fitness pleiotropy were examined, in order to determine their relative importance in influencing fitness pleiotropy. Partial correlation analysis was used to achieve this objective. The partial correlation analysis was restricted to TFs, as the large number of non-TFs may confound our analysis. The results showed that ρ _fitness pleiotropy, CRE | out degree_ = −0.300 (p = 1.3e−05), ρ _fitness pleiotropy, out degree | CRE_ = 0.311 (p = 5.9e−06). The absolute values of the two partial correlations are similar indicating that the strength of contributions of CRE and out-degree to fitness pleiotropy are similar. However, the two partial correlations have different signs indicating that fitness pleiotropy is still negatively associated with CRE when out-degree is controlled and that fitness pleiotropy is still positively associated with out-degree when CRE is controlled. Given that the number of TFs is small, in the following analysis only CRE will be examined.

### Joint Effect of Chromatin Regulation and TATA-Box on Fitness Pleiotropy

The TATA-box is a conserved *cis*-DNA-element found in the eukaryotic promoter regions. Genes are divided into TATA-containing genes and non-TATA- containing genes based on the presence of TATA-box in the promoter region [27]. The TATA-box has been found to be the most important DNA motif for predicting gene expression variation, with TATA-containing genes having significantly higher expression variation than non-TATA-containing genes [13]–[14]. In sharp contrast, TATA-containing genes have lower mean fitness pleiotropy (0.850) than non-TATA-containing genes (1.237), and the difference is highly significant (p = 8.7e−08). In other words, when TATA-containing genes are deleted, low fitness pleiotropy is observed, suggesting that these mutations have a less deleterious effect to the organism.

Furthermore, the presence/absence of TATA-box has been shown to be highly associated with CRE [16]. Therefore, the effect of the TATA-box on fitness pleiotropy, as indicated above, could be explained by CRE if the association between fitness pleiotropy and TATA-box disappears when we control CRE. To confirm this, partial correlation was used to measure the association strength between fitness pleiotropy and CRE/TATA-box after controlling TATA-box/CRE, respectively. The results showed that ρ _fitness pleiotropy, CRE | TATA-box_ = −0.148 (p = 8.9e−18) and ρ _fitness pleiotropy, TATA-box | CRE_ = −0.027 (p = 0.127; treat TATA-containing genes as 1 and non-TATA-containing genes as 0). This indicates that the relationship between fitness pleiotropy and the presence of the TATA motif could be explained by the negative association between fitness pleiotropy and CRE. While interesting, because TATA-containing genes are only about 20% of all yeast genes, we will not consider the presence of the TATA-box further.

### The Relationship between Fitness Pleiotropy and Gene Product Centrality as Measured within the Protein Interaction Network: Protein Interaction Degree, Betweenness, and Clustering Coefficient

**Figure 3:**
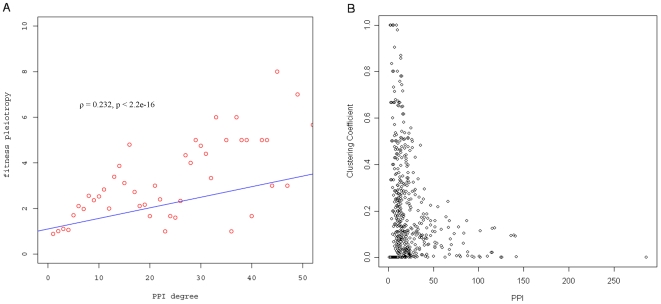
The relationship between fitness pleiotropy and PPI degree (A) and between CC and PPI degree (B). A) The fitness pleiotropy is positively correlated with protein physical interaction (PPI) degree. The Spearman's rank correlation is used to measure the relationship between fitness pleiotropy and PPI degree (ρ = 0.232, p<2.2e−16). Note that only less than 1% of protein has PPI degree higher than 50 (data not shown). The labels are the same as those in Figure 2. B) The scatter plot of the relationship between clustering coefficient and PPI degree. The Spearman correlation coefficient ρ is 0.643 (p<2.2e−16).

The physical interactions between proteins form a protein interaction network. In this network, each protein is a node, and the physical interaction between proteins is an edge. The physical protein interaction degree (PPI degree) is defined as the number of interaction partners for each protein. Since protein interactions play a central role in protein function, proteins with high PPI degree may be involved in more biological processes. Thus, we also expect that genes that encode such proteins will have high fitness pleiotropy. As shown in Figure 3A, as PPI degree increases, fitness pleiotropy of the gene also increases (ρ = 0.232, p<2.2e−16). This result is consistent with the findings of He and Zhang [11] and Yu et al. [12], where they found a relatively weak, yet significant positive association between fitness pleiotropy and PPI degree, using different datasets. The positive association between fitness pleiotropy and PPI degree indicates that when a gene with a high PPI degree is deleted, the functions of many proteins that interact with this protein are likely to be affected, resulting in changes in overall fitness, under different growth conditions. Hence, the importance of a gene with respect to fitness increases with the gene product's PPI degree. The findings are also consistent with previous results that showed that the essential genes, that have the highest fitness pleiotropy, tend to have products with higher physical interaction degrees (in our dataset, p = 1.4e−4) [2], [28].

In this study, a gene's product is considered central (gene centrality) based on a high PPI degree and two other measures: betweenness (BW) and clustering coefficient (CC). First, BW of a target protein is calculated by the fraction of shortest paths that pass through the target protein between any pair of proteins. It thus measures the frequency of target protein usage when the signal is transmitted between two proteins. Yu et al. [28] showed that PPI degree is a better predictor of protein essentiality than BW in a protein interaction network, although the probability of a protein being essential increases with BW.

Here it was examined whether the fitness pleiotropy of a non-essential gene increases with BW. Fitness pleiotropy is significantly positively associated with BW (ρ = 0.178, p<2e−16). PPI degree and BW are also highly correlated with a Spearman correlation of ρ = 0.893 in our dataset. These findings indicate, however, that the high correlation between fitness pleiotropy and BW may be explained by the high correlation between fitness pleiotropy and PPI degree. To determine if this is true, the partial correlation between fitness pleiotropy and PPI degree with BW controlled (ρ_fitness pleiotropy, PPI degree|BW_  = 0.169, p = 1.7e−20) was examined. When PPI degree is controlled, the partial correlation between fitness pleiotropy and BW is −0.077 (p = 2.6e−05), indicating an absolute value much smaller than the partial correlation between fitness pleiotropy and PPI degree when BW is controlled. Note that the sign of ρ_fitness pleiotropy, BW| PPI degree_ is the reverse of the sign of ρ_fitness pleiotropy, BW_. These results indicate that PPI degree is a better predictor of fitness pleiotropy than BW, because the partial correlation between fitness pleiotropy and BW is minimal when PPI degree is controlled. This finding is consistent with the results of Yu et al. [28] that PPI degree is a better predictor of essentiality than BW. Therefore, we will not consider BW in the studies presented below.

Second, the clustering coefficient (CC) for the non-essential genes was examined. Proteins within complexes have higher CC values than other proteins. Since proteins within complexes are more likely to be essential [3], it is also hypothesized that fitness pleiotropy for non-essential genes increases with CC. This is demonstrated by the positive correlation with fitness pleiotropy and CC (ρ = 0.243, p<2.2e−16). Although there is also a high correlation between PPI degree and CC (ρ = 0.643, p<2.2e−16, Figure 3B), this correlation is not as strong as the correlation between PPI degree and BW (ρ = 0.893).

**Figure 4:**
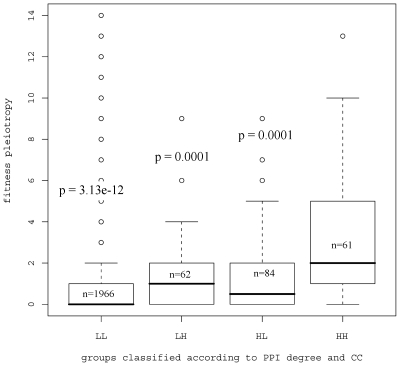
The influence of PPI degree and CC on fitness pleiotropy. Fitness pleiotropy for four different groups of proteins classified according to PPI degree and CC: LL (PPI degree < = 3, CC< = 0); LH (PPI degree < = 3, CC> = 0.4); HL (PPI degree > = 6, CC< = 0); HH (PPI degree > = 6, CC> = 0.4). P-values are given to test the hypothesis that the median fitness pleiotropy in LL, LH, and HL is lower than that in the HH group, respectively. The value of n in the box is the number of genes for each group.

To determine how PPI degree and CC interact to influence fitness pleiotropy, the genes were divided into four groups based on the measurement of PPI degree and CC: low PPI degree, low CC (LL); high PPI degree, low CC (HL); low PPI degree, high CC (LH), and high PPI degree, high CC (HH). Proteins with CC of 0 (76% of the genes) and those with CC of at least 0.4 (5% of the genes) were classified as low CC and high CC, respectively. We chose a low threshold of PPI degree so that the fraction of proteins with low PPI degree is closest to the fraction of proteins with low CC. This resulted in a low PPI degree threshold of 3 (70% of the genes). The upper PPI degree threshold was chosen so that the fraction of proteins with high PPI degree is closest to 20%, which gave a threshold of 6 (18% of the genes). Only about 2% of nonessential gene products are classified in the group having high PPI degree and high CC, whereas most nonessential gene products belong to the group with low PPI and low CC. Figure 4 gives the box plot for the fitness pleiotropy within each group. The results indicate that genes with products of high PPI degree and high CC tend to have the highest fitness pleiotropy. Similar results were obtained when other thresholds were used to partition the proteins into four groups (data not shown).

One explanation for this phenomenon is that proteins with high PPI degree and high CC tend to form complexes that frequently underlie important biological processes, and thus are important for fitness. Inspection of the data leads to the identification of genes with products that function in complexes that underlie important biological processes. For example, *COG7* (PPI = 8, CC = 0.43 and fitness pleiotropy = 6) encodes a component of the cytosolic Golgi tethering complex that functions to mediate fusion of transport vesicles to Golgi compartments [24]. Another example is *CDC10* (PPI = 8, CC = 0.5 and fitness pleiotropy = 7), which encodes a component of the septin ring of the mother-bud neck that is required for cytokinesis [24]. The studies of gene centrality presented here suggest that fitness pleiotropy in nonessential genes increases with PPI degree, BW or CC. PPI degree is a better predictor than BW, and PPI degree may interact with CC influencing fitness pleiotropy.

### The Influence of Gene Expression Variation on the Relationship between Fitness Pleiotropy and PPI Degree, CC and CRE

Many of the gene characteristics measured influencing fitness pleiotropy identified in this study coincide with those influencing gene expression variation, such as CRE, presence/absence of TATA-box, and PPI degree [13]–[16]. Therefore, a natural question that arises is whether fitness pleiotropy can be completely explained by gene expression variation or not. If fitness pleiotropy can be completely explained by gene expression variation, a direct relationship between gene expression variation and fitness pleiotropy could be inferred. Accordingly, the gene expression variation data from a previous study was examined [25], to determine if there is a relationship between fitness pleiotropy and gene expression variation. As shown in the scatter-plot in Figure 2A, there is, indeed, a high correlation between fitness pleiotropy and gene expression variation (ρ = −0.151, p<2.2e−16), but the absolute correlation coefficient is relatively low, indicating that expression variation may only explain a small fraction of fitness pleiotropy.

Genes with fitness pleiotropy of at least 4 (top 11% of the all the genes) (the threshold 4 was chosen so that the fraction of high fitness pleiotropy genes is closest to 10%) and gene expression variation of at least 2970 (top 10% of the genes) were selected as a set with high fitness pleiotropy and high expression variation (0.4% of the data). Interestingly, we found that this set was enriched with the genes that encode ion transporters (P-value  = 0.00019 indicated by FunSpec [29]), especially heavy metal ion transporters, including the iron transporter genes *ftr1*, *fet3* and *ctr1*. Given that iron plays a vital role in many important processes, such as electron transfer, oxygen transport, and DNA synthesis, a deletion of an ion transporter gene is very likely to affect fitness. In *S. cerevisiae*, iron level is primarily mediated by a plasma membrane iron transport system, including products encoded by *ftr1*and *fet3*. Additionally, it was found that expression of the genes that encode the iron transporters are regulated according to iron need in the cell [30]–[31]. Therefore, some genes with high gene expression variation also tend to have high fitness pleiotropy.

Genes (15% of the data) with low fitness pleiotropy (equal to 0) and low expression variation (no greater than 800, low 22% of the genes) were also identified. It should be noted that 60% of the genes in this set encode proteins that have unknown biological function. The set also included genes such as *pex7*, *pex10*, *pex4*, *pex6*, and *pex15*, that encode products involved in peroxisome organization and biogenesis; a high number of these genes encode proteins involved in importing other proteins into the peroxisomal matrix [24]. The genes show low gene expression variation, perhaps because their expression is not influenced by environmental conditions. The low fitness pleiotropy (i.e., 0) suggests that a defect in the biological process that these genes underlie might not affect cell growth significantly. These findings also suggest that the negative correlation between gene expression variation and fitness pleiotropy is not strong and cannot describe some groups of genes.

The partial correlation between fitness pleiotropy and CRE, PPI degree, and CC, were examined by controlling gene expression variation. The results are given in Table 1. For comparison, we also give the correlation between fitness pleiotropy and CRE, PPI degree, and CC when gene expression variation is not controlled. The absolute partial correlation coefficient between fitness pleiotropy and CRE when gene expression variation is controlled is much smaller than that when gene expression variation is not controlled. This result suggests that the association between fitness pleiotropy and CRE can be partially, but not completely, attributed to the association between fitness pleiotropy and gene expression variation. On the other hand, the partial correlation coefficients between fitness pleiotropy and PPI degree, and CC when gene expression is controlled are all similar to the corresponding correlation without controlling gene expression variation indicating that these measurements contribute to fitness pleiotropy independent of expression variation.

**Table 1:** Correlation between fitness pleiotropy and each measurement when expression variation is either controlled or not.

measurement		ρ	p value
CRE	without expression variation controlled	−0.172	<2.2e−16
	with expression variation controlled	−0.112	2.6e−10
PPI degree	without expression variation controlled	0.232	<2.2e−16
	with expression variation controlled	0.225	<2.2e−16
CC	without expression variation controlled	0.243	<2.2e−16
	with expression variation controlled	0.229	<2.2e−16

When gene expression variation is controlled, ρ is partial Spearman's correlation coefficient and p-value is based on null hypothesis test that there is no statistically significant relationship between fitness pleiotropy and each measurement after controlling gene expression variation, i.e., the relationship between fitness pleiotropy and each measurement is explained by gene expression variation.

Based on this result, we next asked what biological mechanism underlies the correlation between fitness pleiotropy and expression variation. In order to answer this question, we studied the partial correlation between fitness pleiotropy and gene expression variation when CRE, PPI degree, or CC is controlled, respectively (see Table 2). When CRE is controlled, fitness pleiotropy and gene expression variation are no longer associated indicating that CRE plays key roles in both fitness pleiotropy and gene expression variation. Thus, CRE can be considered as the key underlying latent variable that controls both fitness pleiotropy and expression variation resulting in their correlation, and fitness pleiotropy and gene expression are independent when CRE is controlled.

**Table 2:** Partial Spearman's correlation between fitness pleiotropy and expression variation when each measurement is controlled.

	ρ	p value
ρ _fitness pleiotropy, expression variation | CRE_	−0.020	0.2662
ρ _fitness pleiotropy, expression variation | PPI degree_	−0.144	3.4e−14
ρ _fitness pleiotropy, expression variation | CC_	−0.143	5.5e−14

ρ is Spearman's correlation coefficient and p-value is based on null hypothesis test that there is no statistically significant relationship between fitness pleiotropy and gene expression variation after controlling CRE, PPI degree or CC, i.e., the relationship between fitness pleiotropy and gene expression is explained by CRE, PPI degree or CC.

### Joint Analysis of PPI Degree, CC and CRE on Fitness Pleiotropy

These findings indicated that the gene characteristics that are significantly associated with fitness pleiotropy are CRE, PPI degree, and CC for the nonessential *S. cerevisiae* genes. Fitness pleiotropy increases with PPI degree and CC, while it decreases with CRE. We also found that, although the presence of TATA-box influences fitness pleiotropy, this phenomenon can be explained by high CRE in TATA-containing genes, which suggests that fitness pleiotropy is no longer associated with TATA-box once CRE is controlled. Based on these findings, the next logical step takes us to a determination of whether such characteristics that were measured collectively explain fitness pleiotropy among all of the nonessential genes. In order to achieve this objective, the partial correlation between fitness pleiotropy and either CRE, PPI or CC measures were examined, when the other two measures are controlled (Table 3). The results show that both CRE and gene centrality (measured by PPI degree and CC) play important roles influencing fitness pleiotropy.

**Table 3:** Partial Spearman's correlation between fitness pleiotropy and CRE, PPI degree or CC.

	ρ	p-value
ρ _fitness pleiotropy, CRE | PPI,CC_	−0.153	3.5e−14
ρ _fitness pleiotropy, PPI | CRE,CC_	0.100	7.5e−07
ρ _fitness pleiotropy, CC | CRE,PPI_	0.114	2.0e−08

Partial Spearman's correlation between fitness pleiotropy and PPI degree refers to Spearman's correlation after controlling CC and CRE. ρ is Spearman's correlation coefficient and p-value is based on null hypothesis test that there is no statistically significant relationship between fitness pleiotropy and each measurement after controlling two other measurements, i.e., such measurement is not significantly associated with fitness pleiotropy in this joint analysis.

In Files S1, S2 and S3, we provide results when MIPS, DIP, or BioGrid protein interaction data sets, and the fitness profiles in Parson et al. [9] or Hillenmeyer et al. [10] were analyzed. It is noted that the association between fitness pleiotropy and PPI degree or CC with/without controlling expression variation when the DIP interaction data was used is much weaker compared to the corresponding association values when MIPS or BioGrid interaction data set was used. The observation can be explained by the relative smaller number of protein interactions in the DIP data set compared to the other two interaction data sets. The results highlight the importance of using increasingly complete interaction data sets for studying the relationship between fitness pleiotropy and gene characteristics within the protein interaction networks. We also note that significant partial correlation of fitness pleiotropy with CRE controlling for PPI and CC, as well as with PPI controlling for CRE and CC, was replicable when other combinations of fitness profiles and protein interaction data sets were used in the analysis. However, the significant partial correlation between fitness pleiotropy and CC controlling for CRE and PPI can only be observed when MIPS interaction data was used, and was not observed when DIP and BioGrid interaction data sets were analyzed. The observations suggest that the association between fitness pleiotropy and CC can potentially be attributed to the association between fitness pleiotropy with CRE and PPI.

This study provides a systematic analysis of genes and their products' functions that influence fitness pleiotropy, for all of the nonessential genes in *S. cerevisiae*. Within the concept of gene centrality and chromatin regulation, the important characteristics identified are CRE and PPI degree. The inter-relationship between these gene centrality measures and regulation by CRs was also examined with respect to expression variation and fitness pleiotropy. The findings suggest that the potential for a gene to be chromatin regulated, as measured by CRE, and the gene centrality, as measured by PPI degree, significantly affect the corresponding gene's fitness pleiotropy. The results from examining the data based on three independent gene deletion experiments, that examined fitness in 51, 82 and 418 conditions, respectively, are consistent. These consistent results indicate that the conclusions should be generally applicable to many other conditions. However, there are several limitations of this study. Both the protein interaction network and gene regulatory network are incomplete and contain false positive and negative errors. To study the effect of incompleteness of the protein interaction network, we did the same type of analyses using the other two protein interaction data sets: DIP [20] and BioGrid [21], and the results are qualitatively similar (see Files S1, S2 and S3). We used the largest gene regulatory network that is currently available in this study. How our results will change when more complete regulatory network data are available is a question for future studies. The characteristics of genes that were studied in this paper include PPI degree, BW, CC, CRE, TATA-box, etc are highly correlated. We used partial correlation analysis to study how these characteristics interact to affect fitness pleiotropy. More advanced methods such as pathway analysis or Bayesian network analysis may uncover more complex relationships among these characteristics and how they interact to influence fitness pleiotropy, a topic for further study.

## Materials and Methods

### Phenotypic Profiles

Three fitness profiles of *S. cerevisiae* deletion strains, which measured the changes of growth rate when nonessential genes were deleted under various conditions were used [8]–[10]. In the main text, the quantitative profile for yeast homozygous deletion strains with each of 4277 genes deleted under 51 conditions were used [8]. When duplicate measures of growth rate for strains with the same deleted genes were available, the average change in growth rate was used in our analysis. A total of 10 genes have duplicate measures, and the results are essentially the same if these genes had been removed in the analysis (data not shown). The refined data were normalized under each condition to a standard normal distribution. To exclude the biological dependency between these 51 conditions, the conditions were classified into 31 groups based on their different effects on the phenotype using two-way clustering [8]. The conditions in the same group have a similar phenotypic profile that was measured by Pearson's correlation coefficient by Brown et al. [8]. The 31 groups are as follows: AAPO,H2O2; Alk.5g,Alk.15g; Bleo,HygB; Cis1,Cis4,Oxa; CPTa,CPTc; ActD,Dox; Gal.5g,Gal.15g; AntA,GlyE; Ida, TPZ;Mech,MMC; Min.5g,Min.15g;NaCl.5g,NaCl.15g; Nys.5g,Nys.15g; Sorb.5g,Sorb.15g; Trp,Thr,Lys,SC; UVB,UVC,IR; and the remaining with each condition as one group. The deletion strain with growth rate change less than -2 (2 standard deviation) is defined as having significant growth defect under the specific condition. A deletion strain has a growth defect under a group of conditions if the deletion strain shows growth defect under at least one of the conditions in this group. The fitness profile data contain the growth rate of yeast haploid deletion strains of 4111 nonessential genes under 82 conditions [9], growth rate of yeast homozygous deletion strains of 4742 nonessential genes under 418 conditions [10], and the details are given in the Files S2 and S3. The fitness pleiotropy measures based on the three phenotype profiles are strongly correlated (See Table 7 in File S1).

### Protein Interaction Networks

The yeast protein interaction data from three different data sources were downloaded: MIPS [19], DIP [20], and BioGrid [21]. The MIPS (Munich Information Center for Protein Sequences) [19] dataset (version: PPI_18052006.tab) contains 11,124 protein physical interactions involving 4,404 proteins. The DIP core interaction dataset [20] (version: ScereCR20070107) contains 5,738 protein interactions involving 2,161 proteins. The DIP core interactions were assessed by a number of quality tests and are supposedly highly reliable [32]. The BioGrid [21] dataset (version 2.0.34) contains 59,317 protein physical interactions involving 5,054 proteins. Previous studies have shown that the MIPS interaction dataset has relatively high reliability compared to other data sources [33]. Therefore, our efforts were concentrated on the results based on MIPS. The results based on DIP and BioGrid are presented as Files S1, S2 and S3. For a given protein interaction dataset, the protein physical interaction (PPI) degree was calculated. The betweenness (BW), and the clustering coefficient (CC) were calculated using the software Pajek 1.20 [34]. Pajek is a software package for large network analysis and visualization.

### Regulatory Network

Transcription factors (TFs) influence the expression of downstream genes. Hu et al. [22] constructed a regulatory network using 263 TF knockout profiles. We used a directed edge from a TF to a gene if the expression of the gene was significantly changed when the TF was knocked out. Note that this regulatory network represents indirect relationship, not necessarily direct regulation. The out-degree of a TF is the number of genes that the TF regulates in this network, while the in-degree is the number of TFs regulating a specific gene in this network.

### Expression Compendium of Chromatin Regulators

To study the effects of chromatin regulation on fitness pleiotropy, the expression compendium of chromatin regulators assembled previously, was used [23]. We removed the expression data under perturbations of TATA binding protein (TBP), histone proteins (H3 and H4), proteins with unknown chromatin regulation activities, as well as comparative perturbations, because they do not represent perturbations of chromatin regulators. Finally, we obtained a reduced dataset of expression profiles for 116 perturbations of chromatin modifiers, Histone mehtyltransferase, acetyltransferases and deacetyltransferases, silencing factors, ubiquitinating, deubiquitinating enzymes and ATPase. We further checked the percentage of missing values for each gene under 116 perturbations. If a gene had more than 10% (i.e., 12) missing values, we excluded it in the final refined data. We normalized the refined data under each perturbation to a standard normal distribution and calculated chromatin regulator effect (CRE) as the average of absolute value of logarithm of the gene expression changes across 116 perturbations, which is the same as [16].

### TATA-Containing Genes

A TATA-box is a DNA sequence motif (*cis*-element) found in the promoter region of most eukaryotic genes. The TATA consensus sequence was identified as TATA(A/T)A(A/T)(A/G) [27]. The relationship between yeast genes and the TATA box was downloaded from [27].

### Statistical Analysis

In our dataset, fitness pleiotropy is a discrete response variable. To measure the relationship between fitness pleiotropy and each measurement, we used a non-parametric Spearman's rank correlation with corresponding statistical significant test since the assumptions of parametric methods, such as linear regression or ordinal logistic regression, are not satisfied. Spearman's rank correlation is used to discover the linear association between two variables, and its corresponding test has no distribution assumptions for the variables. In the joint analysis, non-parametric Spearman partial correlation and the corresponding significant test are used to measure which measurement is most important in influencing fitness pleiotropy. We also used Spearman partial correlation to find the relative importance of measurements influencing fitness pleiotropy. For example, if we want to know which of measurement y or z has a stronger association with x, we compare the value of ρ_x,y|z_ and ρ_x,z|y_. The bigger value means the stronger association. ρ_x,y|z_ means partial correlation between x and y after controlling z.

The first order partial correlation is defined as:


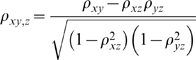
 where ρ_xy_ is the correlation between x and y.

The second order partial correlation is defined as:


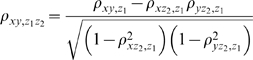
 where ρ_xy,z_ is the partial correlation between x and y after controlling z. It is implemented by SAS 9.0 (http://www.sas.com/technologies/bi/appdev/base/).

To provide visualization of the relationship between fitness pleiotropy and each measurement, we used linear regression to fit the data in the plot. 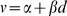
where v is fitness pleiotropy and d is the measurement value. α and β are parameters.

We also used box plots for visualization in our studies. These show the difference in distribution of each variable. The line in the box indicates the median value. The upper edge of the box indicates the 75^th^ percentile, and the lower edge indicates the 25^th^ percentile. The ends of the vertical line indicate the minimum and the maximum values, and the points outside the ends of the vertical line are outliers.

In addition, we used a non-parametric Wilcoxon rank sum test [35] to compare the difference in median for two distributions. The test in our study is a one-side test that is based on the alternative hypothesis that variable A has higher or lower value than variable B.

## Supporting Information

File S1 Provides analysis results based on phenotypic file from Brown et al. [8] and the results with regard to protein interaction degree using DIP [20] and BioGrid [21] data sets.
(0.50 MB DOC)

File S2 Provides analysis results based on phenotypic file from Parsons et al. [9] and the results with regard to protein interaction degree using MIPS [19], DIP [20] and BioGrid [21] data sets.
(1.60 MB DOC)

File S3 Provides analysis results based on phenotypic file from Hillenmeyer et al. [10] and the results with regard to protein interaction degree using MIPS [19], DIP [20] and BioGrid [21] data sets.
(1.15 MB DOC)
