# Supplementary material for: Chromatin Regulation and Gene Centrality Are Essential for Controlling Fitness Pleiotropy in Yeast
Source: PLoS One. 2009 Nov 30;4(11):e8086. doi: 10.1371/journal.pone.0008086 (PMC2778950; doi:10.1371/journal.pone.0008086)
Supplement: File S1 — Provides analysis results based on phenotypic file from Brown et al. [8] and the results with regard to protein interaction degree using DIP [20] and BioGrid [21] data sets. (0.50 MB DOC) [file pone.0008086.s001.doc]

**Supporting Information File S1**

**1. Analysis using Brown et al. [2006] phenotypic data and DIP protein interaction data…..................................................2**

**2. Analysis using Brown et al. [2006] phenotypic data and BioGrid protein interaction data…....................................7**

**3. The correlation among three phenotypic profiles…….12**

**Analysis using Brown et al. [2006] data and DIP protein interaction data**


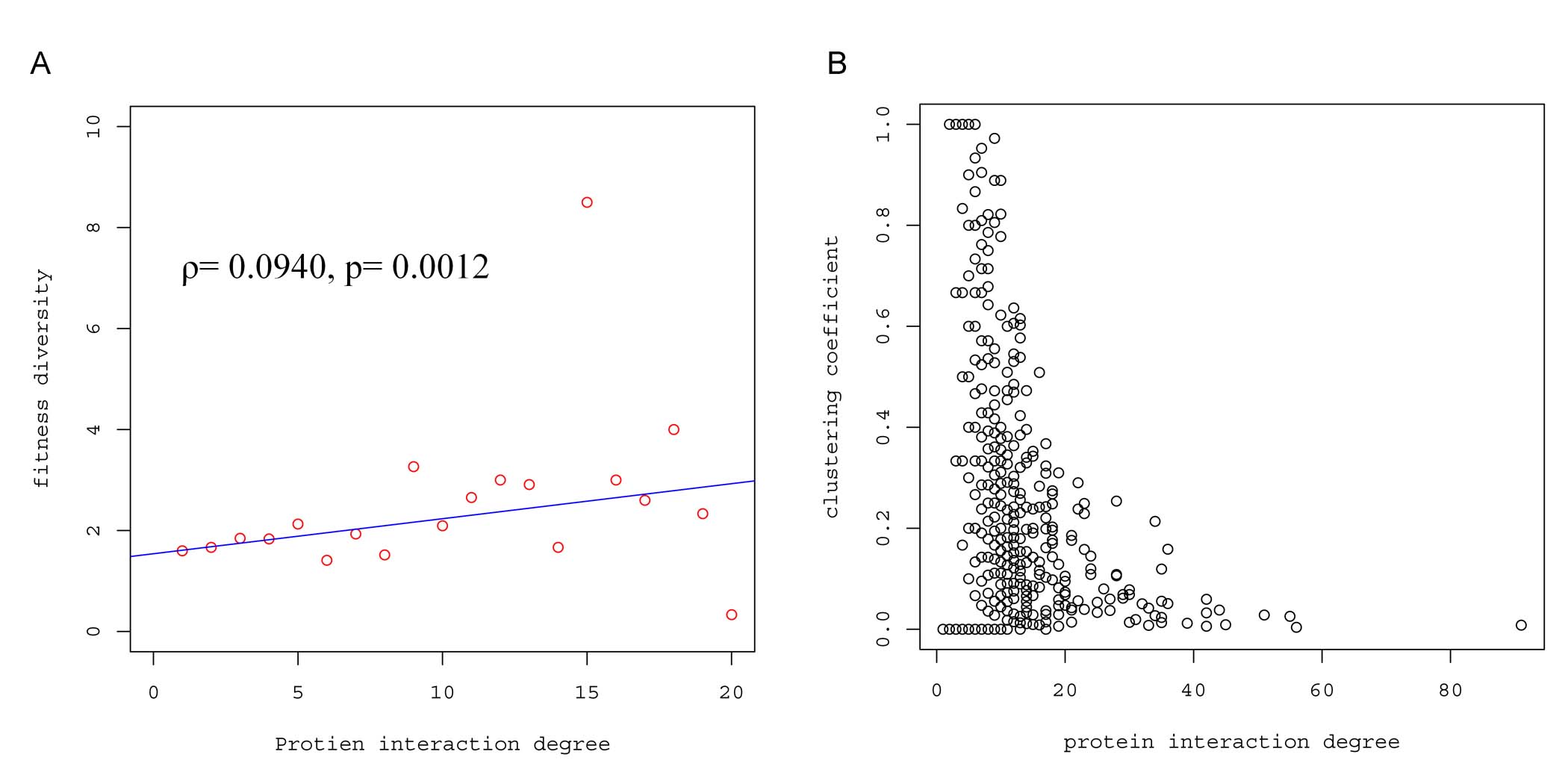


Supplemental Figure 1. The relationship between fitness pleiotropy and protein interaction degree (A) and between CC and protein interaction degree (B). A) The fitness pleiotropy is positively correlated with protein interaction degree. The Spearman’s rank correlation is used to measure the relationship between fitness pleiotropy and protein interaction degree (ρ= 0.094, p=0.0012). The red dots are the mean fitness pleiotropy of the genes, given protein interaction degree. Note that only about 1% of protein has protein interaction degree higher than 0 (data not shown). For visualization, the blue line represents linear regression. B) The scatter plot of the relationship between clustering coefficient and protein interaction degree. The Spearman correlation coefficient ρ is 0.539 (p< 2.2e-16).


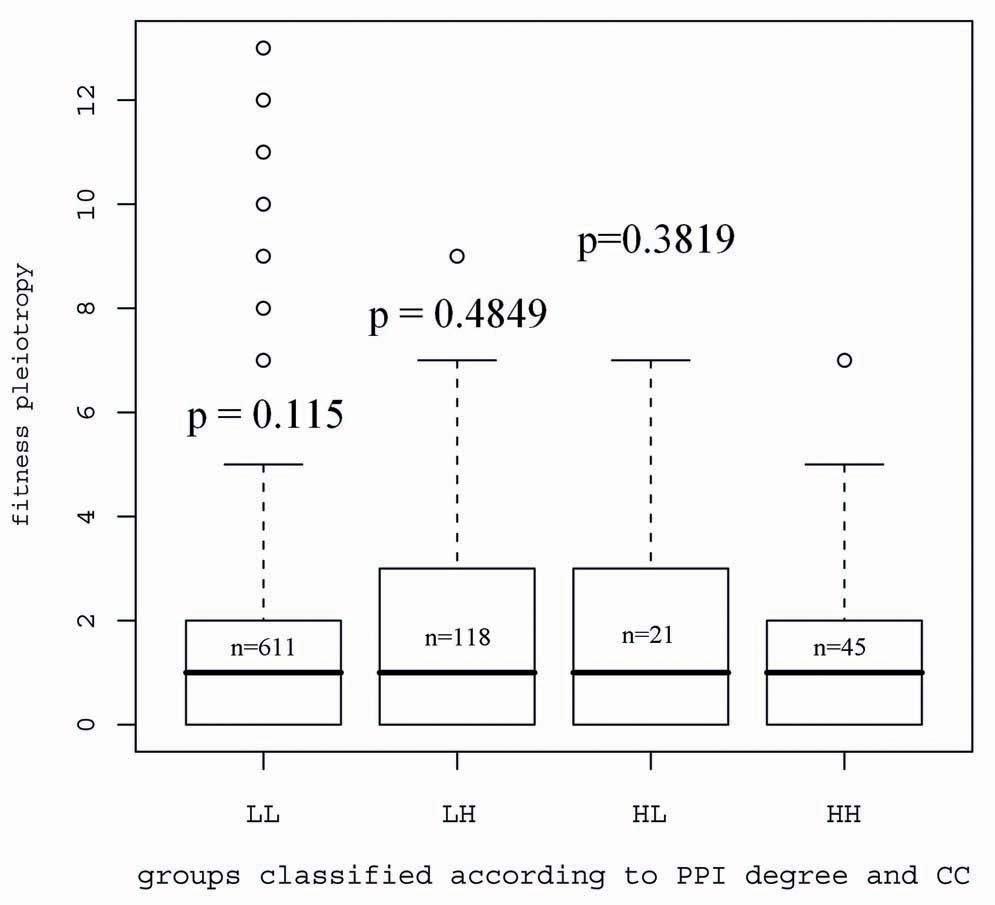


Supplemental Figure 2. Boxplot of fitness pleiotropy for different groups of proteins classified according to protein interaction degree and CC: LL (protein interaction degree <=3, CC<=0); LH (protein interaction degree <=3, CC>=0.4); HL (protein interaction degree >=6, CC<=0); HH (protein interaction degree >=6, CC>=0.4). P-values are given to test the hypothesis that the median fitness pleiotropy in LL, LH, and HL is lower than that in the HH group, respectively. The upper edge of the box indicates the 75^th^ percentile, and the lower edge indicates the 25^th^ percentile. The ends of the vertical line indicate the minimum and the maximum values, and the points outside the ends of the vertical line are outliers. The value of n in the box is the number of genes for each group.

Supplemental Table 1. Spearman’s correlation between fitness pleiotropy and protein interaction degree, CC when gene expression variation is either controlled or not. When gene expression variation is controlled, ρ is partial Spearman’s correlation coefficient and p-value is based on null hypothesis test that there is no statistically significant relationship between fitness pleiotropy and each measurement after controlling gene expression variation, i.e., the relationship between fitness pleiotropy and each measurement is explained by gene expression variation.

| measurement |  | ρ | p value |
| --- | --- | --- | --- |
| Protein interaction degree | without expression variation controlled | 0.094 | 0.001 |
|  | with expression variation controlled | 0.092 | 0.002 |
| CC | without expression variation controlled | 0.095 | 0.001 |
|  | with expression variation controlled | 0.062 | 0.039 |

Supplemental Table 2. Partial Spearman’s correlation between fitness pleiotropy and gene expression variation when protein interaction degree, CC is controlled. ρ is Spearman’s correlation coefficient and p-value is based on null hypothesis test that there is no statistically significant relationship between fitness pleiotropy and gene expression variation after controlling protein interaction degree, CC.

|  | Ρ | p value |
| --- | --- | --- |
| ρ fitness pleiotropy, expression variation | Protein interaction degree | -0.197 | 1.5e-11 |
| ρ fitness pleiotropy, expression variation | CC | -0.191 | 6.3e-11 |

Supplemental Table 3. Partial Spearman’s correlation between fitness pleiotropy and protein interaction degree, CC or CRE. Partial Spearman’s correlation between fitness pleiotropy and protein interaction degree means Spearman’s correlation after controlling CC and CRE. ρ is Spearman’s correlation coefficient and p-value is based on null hypothesis test that there is no statistically significant relationship between fitness pleiotropy and each measurement after controlling another two measurements.

|  | Ρ | p-value |
| --- | --- | --- |
| ρ fitness pleiotropy, CRE | Protein interaction degree,CC | -0.188 | 1.0e-09 |
| ρ fitness pleiotropy, Protein interaction degree | CRE,CC | 0.052 | 0.094 |
| ρ fitness pleiotropy, CC | CRE, Protein interaction degree | 0.032 | 0.301 |

**Analysis using Brown et al. [2006] data and BioGrid protein interaction data**


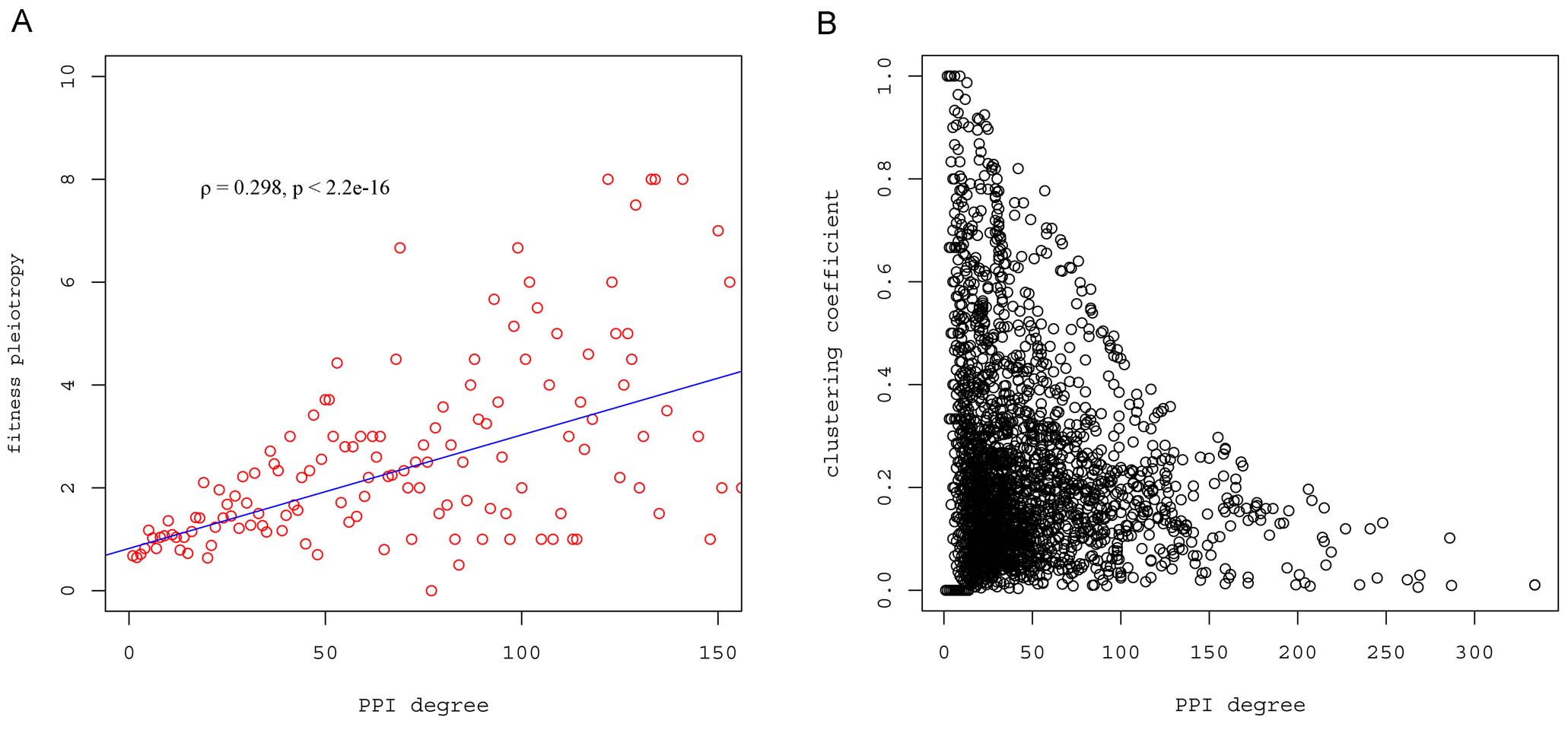


Supplemental Figure 3. The relationship between fitness pleiotropy and PPI degree (A) and between CC and PPI degree (B). A) The fitness pleiotropy is positively correlated with protein physical interaction (PPI) degree. The Spearman’s rank correlation is used to measure the relationship between fitness pleiotropy and PPI degree (ρ= 0.298, p< 2.2e-16). The red dots are the mean fitness pleiotropy of the genes, given PPI degree. Note that only about 1% of protein has PPI degree higher than 150 (data not shown). For visualization, the blue line represents linear regression. B) The scatter plot of the relationship between clustering coefficient and PPI degree. The Spearman correlation coefficient ρ is 0.462 (p< 2.2e-16).


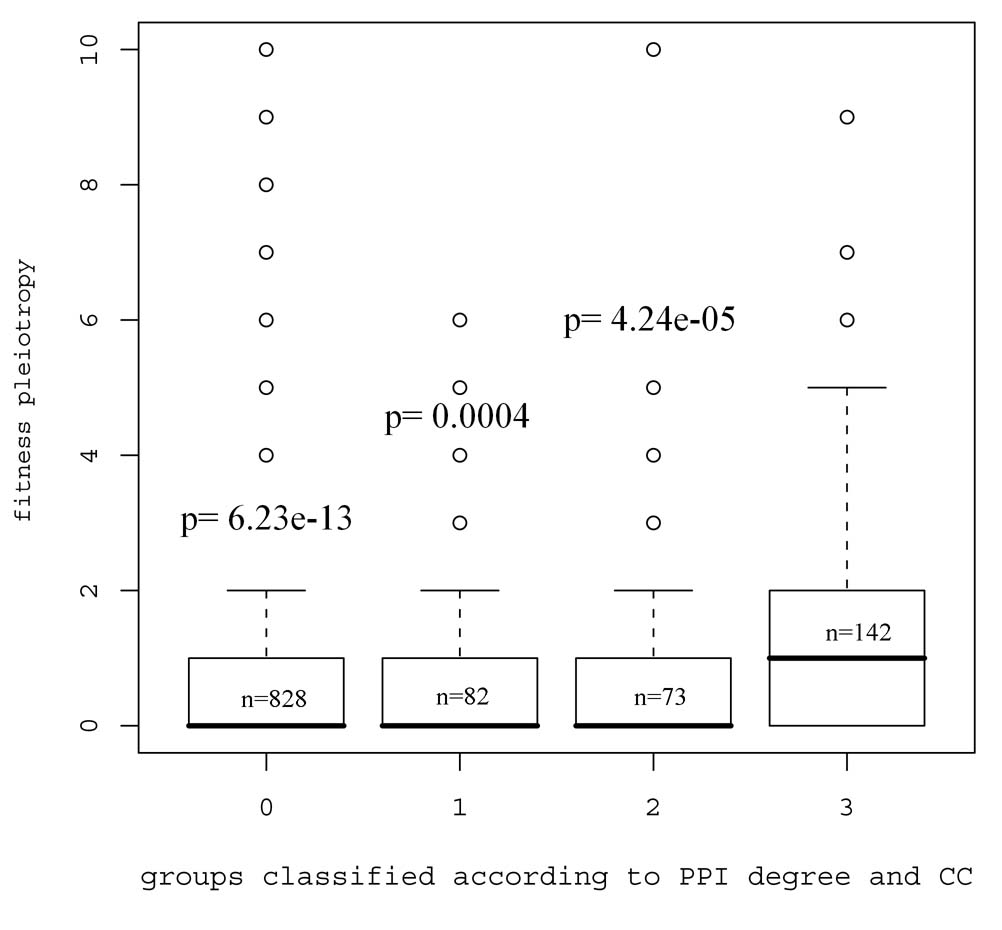


Supplemental Figure 4. Boxplot of fitness pleiotropy for different groups of proteins classified according to PPI degree and CC: LL (PPI degree <=3, CC<=0); LH (PPI degree <=3, CC>=0.4); HL (PPI degree >=6, CC<=0); HH (PPI degree >=6, CC>=0.4). P-values are given to test the hypothesis that the median fitness pleiotropy in LL, LH, and HL is lower than that in the HH group, respectively. The upper edge of the box indicates the 75^th^ percentile, and the lower edge indicates the 25^th^ percentile. The ends of the vertical line indicate the minimum and the maximum values, and the points outside the ends of the vertical line are outliers. The value of n in the box is the number of genes for each group.

Supplemental Table 4. Spearman’s correlation between fitness pleiotropy and PPI degree, CC when gene expression variation is either controlled or not. When gene expression variation is controlled, ρ is partial Spearman’s correlation coefficient and p-value is based on null hypothesis test that there is no statistically significant relationship between fitness pleiotropy and each measurement after controlling gene expression variation, i.e., the relationship between fitness pleiotropy and each measurement is explained by gene expression variation.

| measurement |  | ρ | p value |
| --- | --- | --- | --- |
| PPI degree | without expression variation controlled | 0.298 | < 2.2e-16 |
|  | with expression variation controlled | 0.292 | < 2.2e-16 |
| CC | without expression variation controlled | 0.168 | < 2.2e-16 |
|  | with expression variation controlled | 0.145 | 2.3e-16 |

Supplemental Table 5. Partial Spearman’s correlation between fitness pleiotropy and gene expression variation when PPI degree, CC is controlled. ρ is Spearman’s correlation coefficient and p-value is based on null hypothesis test that there is no statistically significant relationship between fitness pleiotropy and gene expression variation after controlling PPI degree, CC.

|  | Ρ | p value |
| --- | --- | --- |
| ρ fitness pleiotropy, expression variation | PPI degree | -0.119 | 1.4e-11 |
| ρ fitness pleiotropy, expression variation | CC | -0.128 | 4.0e-13 |

Supplemental Table 6. Partial Spearman’s correlation between fitness pleiotropy and PPI degree, CC or CRE. Partial Spearman’s correlation between fitness pleiotropy and PPI degree means Spearman’s correlation after controlling CC and CRE. ρ is Spearman’s correlation coefficient and p-value is based on null hypothesis test that there is no statistically significant relationship between fitness pleiotropy and each measurement after controlling another two measurements.

|  | ρ | p-value |
| --- | --- | --- |
| ρ fitness pleiotropy, CRE | PPI,CC | -0.126 | 1.2e-11 |
| ρ fitness pleiotropy, PPI | CRE,CC | 0.239 | 2.8e-3 |
| ρ fitness pleiotropy, CC | CRE,PPI | -0.006 | 0.7626 |

Supplemental Table 7. The Spearman’s rank correlation among three phenotypic profiles used for our analysis.

|  | Brown et al. [2006] | Parson et al. [2006] | Hillenmeyer et al. [2008] |
| --- | --- | --- | --- |
| Brown et al. [2006] |  | ρ= 0.38, p<2e-16 | ρ= 0.66, p<2e-16 |
| Parson et al. [2006] |  |  | ρ= 0.39, p<2e-16 |
| Hillenmeyer et al. [2008] |  |  |  |
