# Supplementary material for: Chromatin Regulation and Gene Centrality Are Essential for Controlling Fitness Pleiotropy in Yeast
Source: PLoS One. 2009 Nov 30;4(11):e8086. doi: 10.1371/journal.pone.0008086 (PMC2778950; doi:10.1371/journal.pone.0008086)
Supplement: File S3 — Provides analysis results based on phenotypic file from Hillenmeyer et al. [10] and the results with regard to protein interaction degree using MIPS [19], DIP [20] and BioGrid [21] data sets. (1.15 MB DOC) [file pone.0008086.s003.doc]

**Supporting Information File S3**

**1. Analysis using Hillenmeyer et al. [2008] phenotypic data and MIPS protein interaction data….............................................2**

**2. Analysis using Hillenmeyer et al. [2008] phenotypic data and DIP protein interaction data…................................................10**

**3. Analysis using Hillenmeyer et al. [2008] phenotypic data and BioGrid protein interaction data….........................................15**

**Analysis results using Hillenmeyer et al. [2008] data and MIPS protein interaction data**

The Hillenmeyer et al. (2008) dataset contains the growth rate change profile for mutant strains with each of 4742 homozygous nonessential genes deleted under 418 conditions. Conditions include some compounds tested at different concentrations and different time point of collections, temperature change as well as different PH value. These 418 conditions were classified into 158 unique conditions including unique compound, unique temperature change treatment, unique PH treatment. The fitness pleiotropy is defined in the main text.


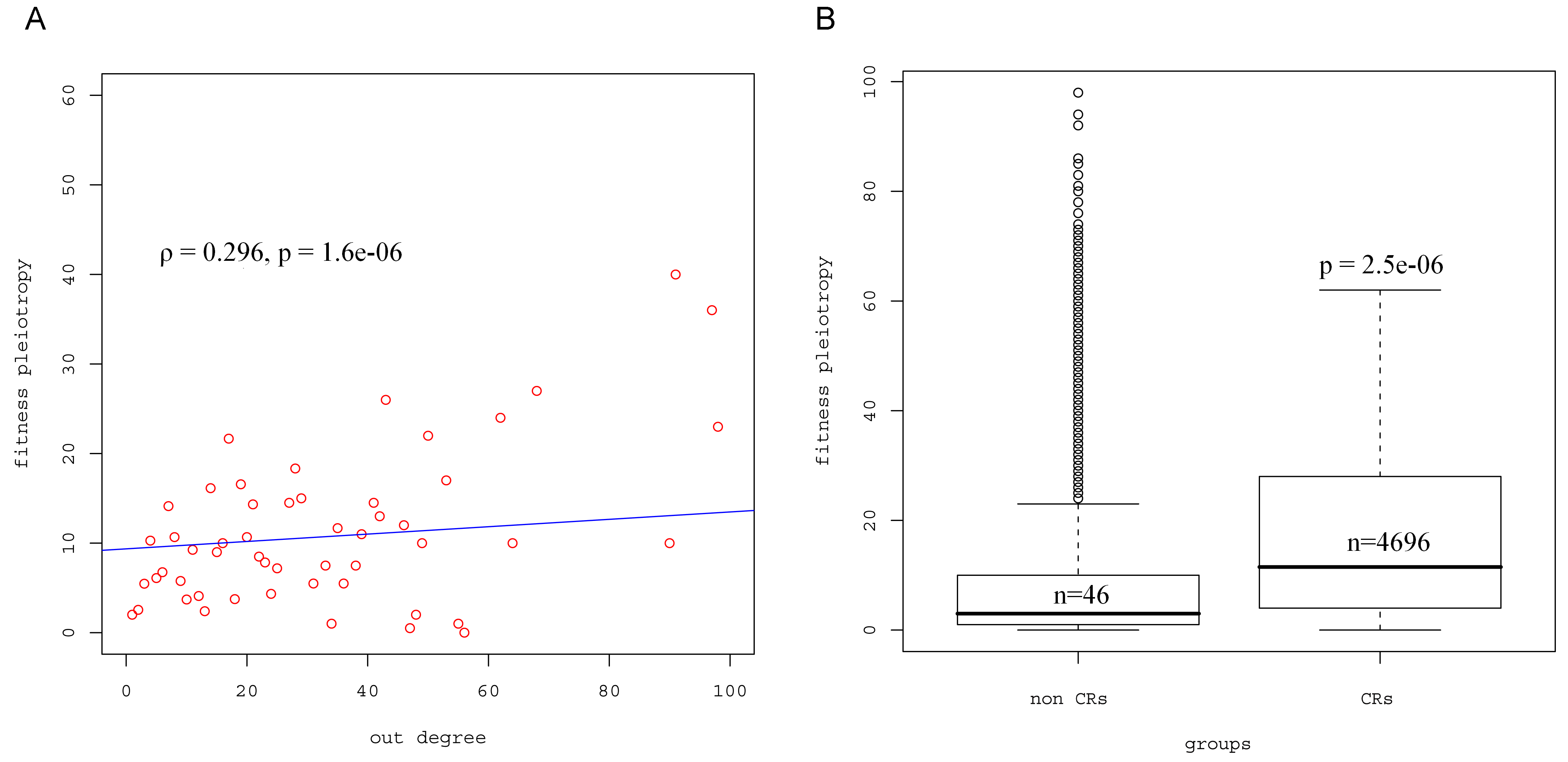


Supplemental Figure 13. The relationship between fitness pleiotropy and different measurements. A) Fitness pleiotropy is positively associated with the number of targeted genes that each TF regulates (ρ= 0.296, p= 1.6e-06). B) Fitness pleiotropy for CRs and non CRs. The line in the box indicates the median value. The upper edge of the box indicates the 75^th^ percentile, and the lower edge indicates the 25^th^ percentile. The ends of the vertical line indicate the minimum and the maximum values, and the points outside the ends of the vertical line are outliers. P-values are given to test the hypothesis that the median fitness pleiotropy for CRs is higher than that for non CRs using non-parametric Wilcoxon rank sum test. The value of n in the box is the number of genes for each group.


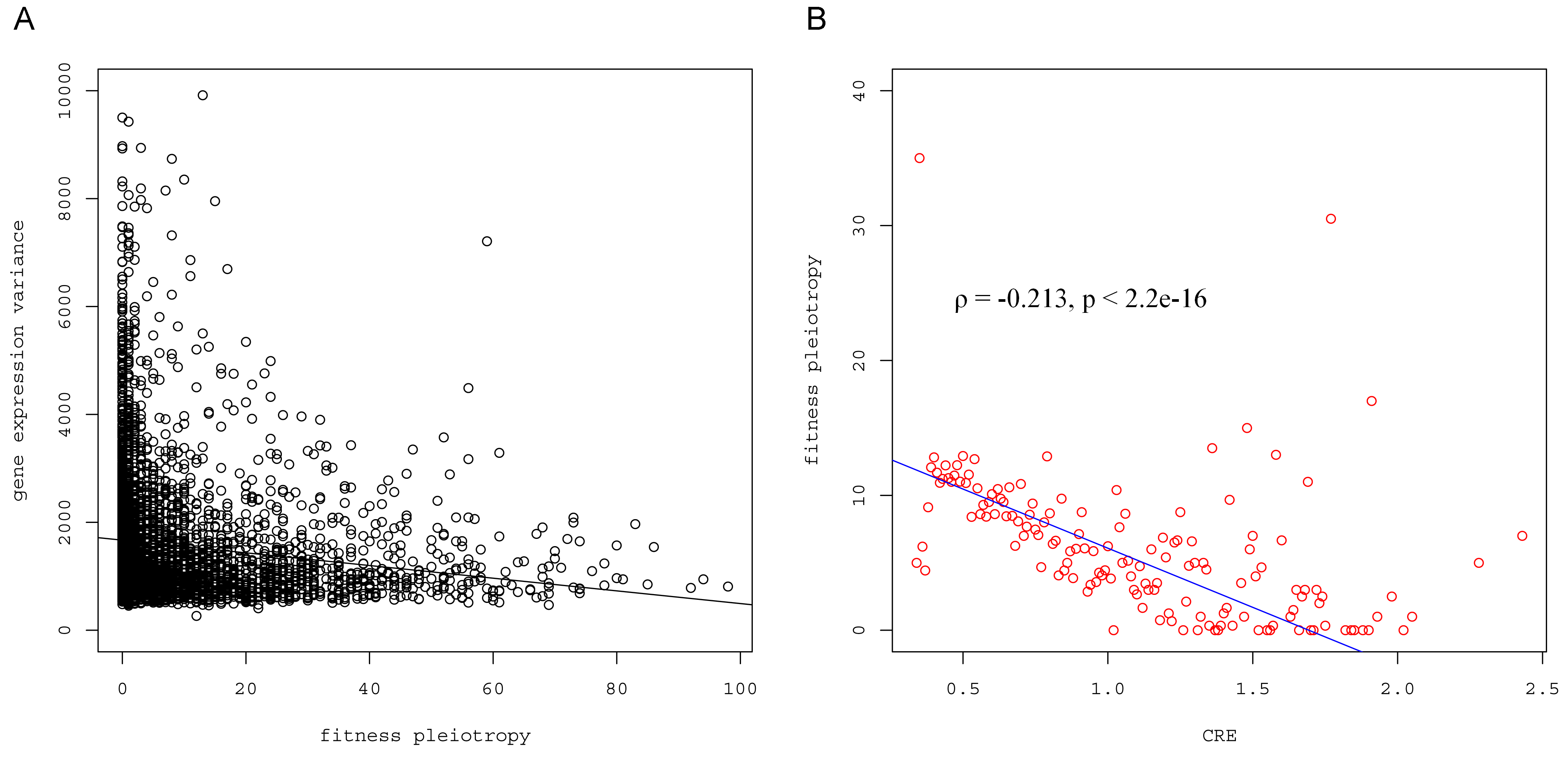


Supplemental Figure 14. The relationship between fitness pleiotropy and different measurements. A) Fitness pleiotropy is significantly negatively associated with gene expression variation (ρ= -0.173, p < 2.2e-16). B) Fitness pleiotropy is negatively associated with chromatin regulatory effect (CRE) (ρ= -0.213, p < 2.2e-16). The labels are the same as those in Supplemental Figure 1.


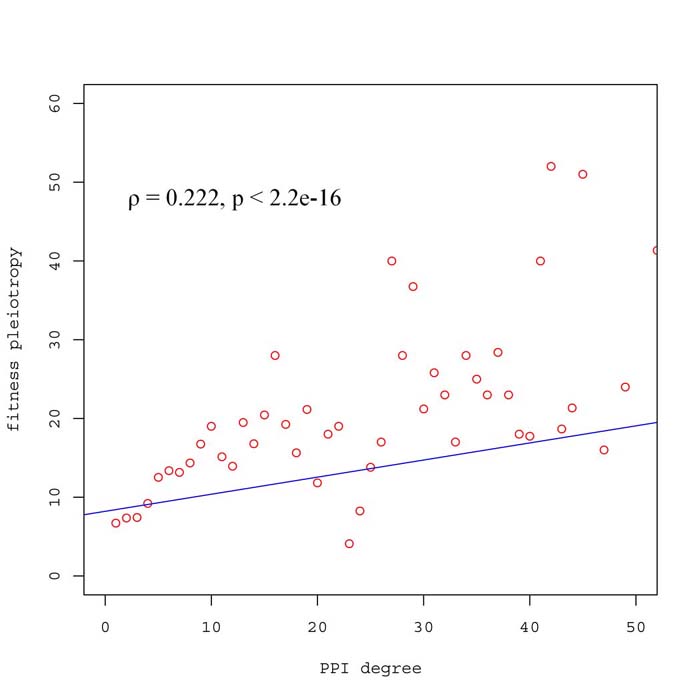


Supplemental Figure 15. The fitness pleiotropy is positively correlated with protein physical interaction (PPI) degree. The Spearman’s rank correlation is used to measure the relationship between fitness pleiotropy and PPI degree (ρ= 0.222, p< 2.2e-16). The red dots are the mean fitness pleiotropy of the genes, given the PPI degree. For visualization, the blue line represents linear regression.


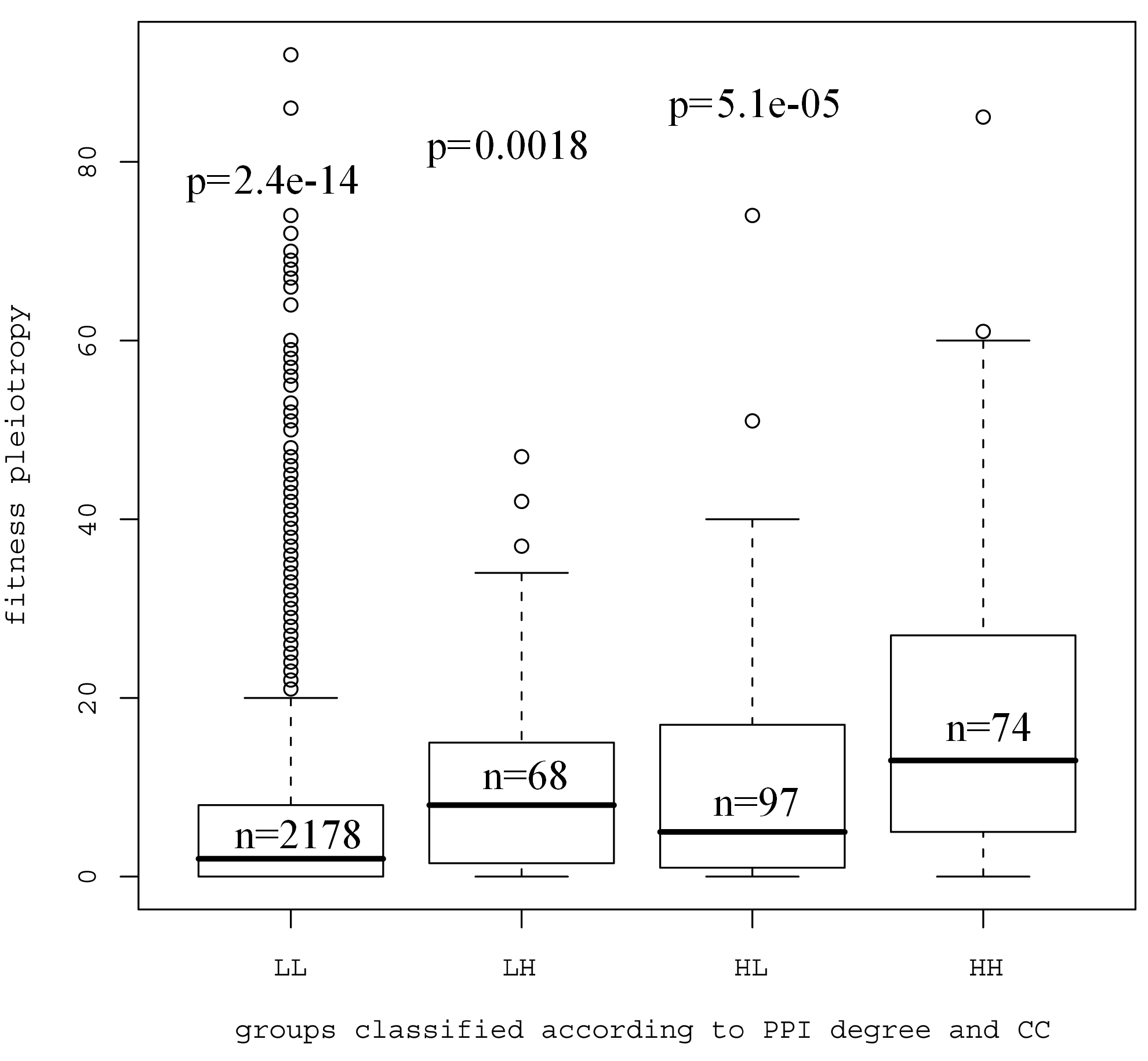


Supplemental Figure 16. The influence of PPI degree and CC on fitness pleiotropy. Fitness pleiotropy for four different groups of proteins classified according to PPI degree and CC: LL (PPI degree <=3, CC<=0); LH (PPI degree <=3, CC>=0.4); HL (PPI degree >=6, CC<=0); HH (PPI degree >=6, CC>=0.4). P-values are given to test the hypothesis that the median fitness pleiotropy in LL, LH, and HL is lower than that in the HH group, respectively. The value of n in the box is the number of genes for each group.

Supplemental Table 17. Correlation between fitness pleiotropy and each measurement when expression variation is either controlled or not. When gene expression variation is controlled, ρ is partial Spearman’s correlation coefficient and p-value is based on null hypothesis test that there is no statistically significant relationship between fitness pleiotropy and each measurement after controlling gene expression variation, i.e., the relationship between fitness pleiotropy and each measurement is explained by gene expression variation.

| measurement |  | ρ | p value |
| --- | --- | --- | --- |
| CRE | without expression variation controlled | -0.213 | < 2.2e-16 |
|  | with expression variation controlled | -0.145 | < 2.2e-16 |
| PPI degree | without expression variation controlled | 0.222 | < 2.2e-16 |
|  | with expression variation controlled | 0.211 | < 2.2e-16 |
| CC | without expression variation controlled | 0.234 | < 2.2e-16 |
|  | with expression variation controlled | 0.216 | < 2.2e-16 |

Supplemental Table 18. Partial Spearman’s correlation between fitness pleiotropy and expression variation when each measurement is controlled. ρ is Spearman’s correlation coefficient and p-value is based on null hypothesis test that there is no statistically significant relationship between fitness pleiotropy and gene expression variation after controlling CRE, PPI degree or CC, i.e., the relationship between fitness pleiotropy and gene expression is explained by CRE, PPI degree or CC.

|  | ρ | p value |
| --- | --- | --- |
| ρ fitness pleiotropy, expression variation | CRE | -0.019 | 0.252 |
| ρ fitness pleiotropy, expression variation | PPI degree | -0.158 | < 2.2e-16 |
| ρ fitness pleiotropy, expression variation | CC | -0.157 | < 2.2e-16 |

Supplemental Table 19. Partial Spearman’s correlation between fitness pleiotropy and PPI degree, CC or CRE. Partial Spearman’s correlation between fitness pleiotropy and PPI degree refers to Spearman’s correlation after controlling CC and CRE. ρ is Spearman’s correlation coefficient and p-value is based on null hypothesis test that there is no statistically significant relationship between fitness pleiotropy and each measurement after controlling two other measurements, i.e., such measurement is not significantly associated with fitness pleiotropy in this joint analysis.

|  | ρ | p-value |
| --- | --- | --- |
| ρ fitness pleiotropy, CRE | PPI,CC | -0.152 | < 2.2e-16 |
| ρ fitness pleiotropy, PPI | CRE,CC | 0.097 | 6.5e-08 |
| ρ fitness pleiotropy, CC | CRE,PPI | 0.107 | 2.9e-09 |

**Analysis results using Hillenmeyer et al. [2008] data and DIP protein interaction data**


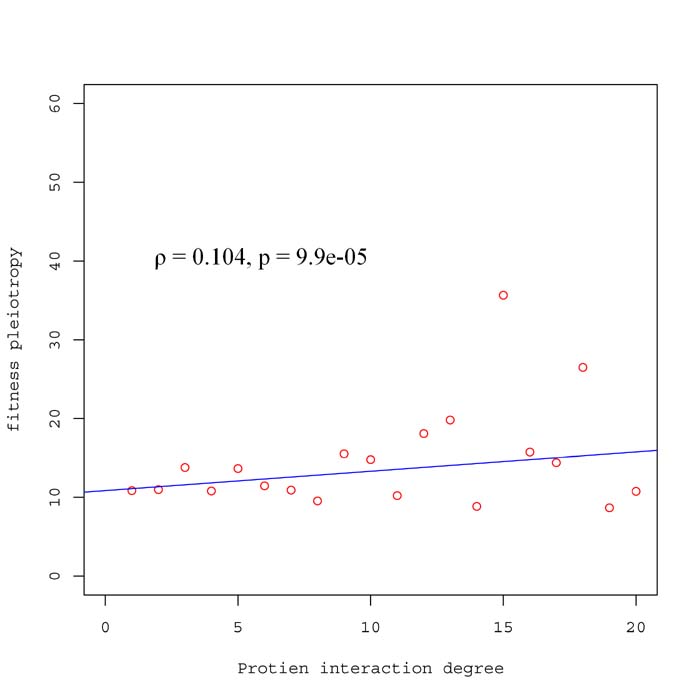


Supplemental Figure 17. The relationship between fitness pleiotropy and protein interaction degree. The fitness pleiotropy is positively correlated with protein interaction degree. The Spearman’s rank correlation is used to measure the relationship between fitness pleiotropy and protein interaction degree (ρ= 0.104, p= 9.9e-05). The red dots are the mean fitness pleiotropy of the genes, given protein interaction degree. Note that only about 1% of protein has protein interaction degree higher than 20 (data not shown). For visualization, the blue line represents linear regression.


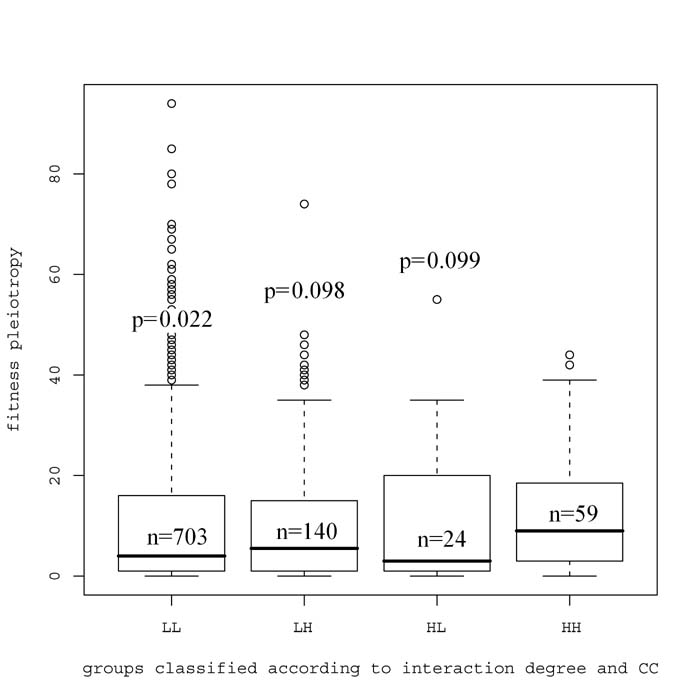


Supplemental Figure 18. Boxplot of fitness pleiotropy for different groups of proteins classified according to protein interaction degree and CC: LL (protein interaction degree <=3, CC<=0); LH (protein interaction degree <=3, CC>=0.4); HL (protein interaction degree >=6, CC<=0); HH (protein interaction degree >=6, CC>=0.4). P-values are given to test the hypothesis that the median fitness pleiotropy in LL, LH, and HL is lower than that in the HH group, respectively. The upper edge of the box indicates the 75^th^ percentile, and the lower edge indicates the 25^th^ percentile. The ends of the vertical line indicate the minimum and the maximum values, and the points outside the ends of the vertical line are outliers. The value of n in the box is the number of genes for each group.

Supplemental Table 20. Spearman’s correlation between fitness pleiotropy and protein interaction degree, CC when gene expression variation is either controlled or not. When gene expression variation is controlled, ρ is partial Spearman’s correlation coefficient and p-value is based on null hypothesis test that there is no statistically significant relationship between fitness pleiotropy and each measurement after controlling gene expression variation, i.e., the relationship between fitness pleiotropy and each measurement is explained by gene expression variation.

| measurement |  | ρ | p value |
| --- | --- | --- | --- |
| Protein interaction degree | without expression variation controlled | 0.104 | 9.9e-05 |
|  | with expression variation controlled | 0.102 | 1.8e-04 |
| CC | without expression variation controlled | 0.081 | 0.002 |
|  | with expression variation controlled | 0.046 | 0.091 |

Supplemental Table 21. Partial Spearman’s correlation between fitness pleiotropy and gene expression variation when protein interaction degree, CC is controlled. ρ is Spearman’s correlation coefficient and p-value is based on null hypothesis test that there is no statistically significant relationship between fitness pleiotropy and gene expression variation after controlling protein interaction degree, CC.

|  | ρ | p value |
| --- | --- | --- |
| ρ fitness pleiotropy, expression variation | protein interaction degree | -0.217 | 4.8e-16 |
| ρ fitness pleiotropy, expression variation | CC | -0.215 | 1.0e-15 |

Supplemental Table 22. Partial Spearman’s correlation between fitness pleiotropy and protein interaction degree, CC or CRE. Partial Spearman’s correlation between fitness pleiotropy and protein interaction degree means Spearman’s correlation after controlling CC and CRE. ρ is Spearman’s correlation coefficient and p-value is based on null hypothesis test that there is no statistically significant relationship between fitness pleiotropy and each measurement after controlling another two measurements.

|  | ρ | p-value |
| --- | --- | --- |
| ρ fitness pleiotropy, CRE | Protein interaction degree,CC | -0.225 | 1.1e-15 |
| ρ fitness pleiotropy, Protein interaction degree | CRE,CC | 0.080 | 0.005 |
| ρ fitness pleiotropy, CC | CRE, Protein interaction degree | -0.005 | 0.862 |

**Analysis results using Hillenmeyer et al. [2008] data and BioGrid protein interaction data**


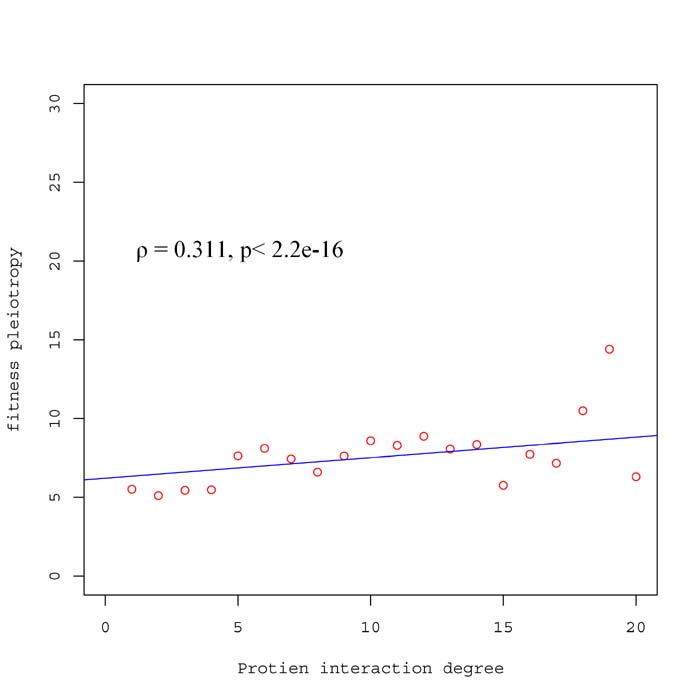


Supplemental Figure 19. The relationship between fitness pleiotropy and protein interaction degree. The fitness pleiotropy is positively correlated with protein interaction degree. The Spearman’s rank correlation is used to measure the relationship between fitness pleiotropy and protein interaction degree (ρ= 0.311, p< 2.2e-16). The red dots are the mean fitness pleiotropy of the genes, given protein interaction degree. Note that only about 1% of protein has protein interaction degree higher than 20 (data not shown). For visualization, the blue line represents linear regression.


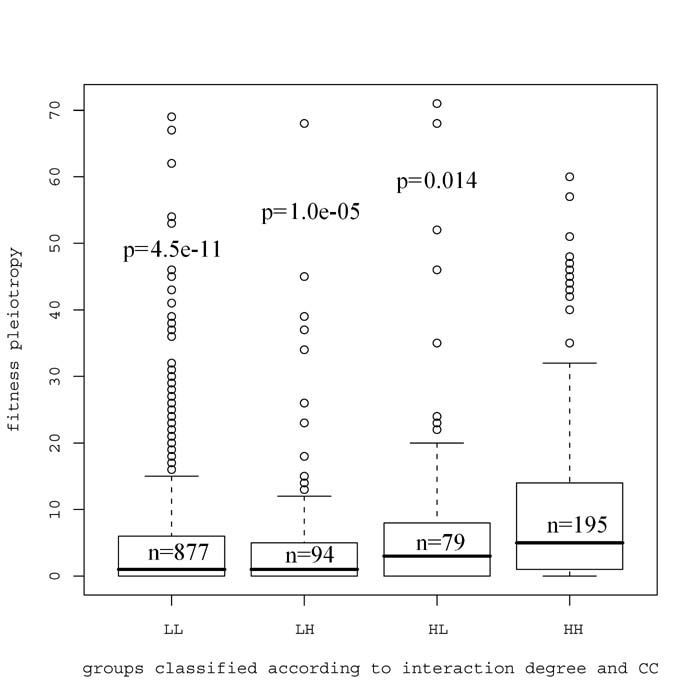


Supplemental Figure 20. Boxplot of fitness pleiotropy for different groups of proteins classified according to PPI degree and CC: LL (PPI degree <=3, CC<=0); LH (PPI degree <=3, CC>=0.4); HL (PPI degree >=6, CC<=0); HH (PPI degree >=6, CC>=0.4). P-values are given to test the hypothesis that the median fitness pleiotropy in LL, LH, and HL is lower than that in the HH group, respectively. The upper edge of the box indicates the 75^th^ percentile, and the lower edge indicates the 25^th^ percentile. The ends of the vertical line indicate the minimum and the maximum values, and the points outside the ends of the vertical line are outliers. The value of n in the box is the number of genes for each group.

Supplemental Table 23. Spearman’s correlation between fitness pleiotropy and PPI degree, CC when gene expression variation is either controlled or not. When gene expression variation is controlled, ρ is partial Spearman’s correlation coefficient and p-value is based on null hypothesis test that there is no statistically significant relationship between fitness pleiotropy and each measurement after controlling gene expression variation, i.e., the relationship between fitness pleiotropy and each measurement is explained by gene expression variation.

| measurement |  | ρ | p value |
| --- | --- | --- | --- |
| PPI degree | without expression variation controlled | 0.311 | < 2.2e-16 |
|  | with expression variation controlled | 0.304 | < 2.2e-16 |
| CC | without expression variation controlled | 0.168 | < 2.2e-16 |
|  | with expression variation controlled | 0.148 | < 2.2e-16 |

Supplemental Table 24. Partial Spearman’s correlation between fitness pleiotropy and gene expression variation when PPI degree, CC is controlled. ρ is Spearman’s correlation coefficient and p-value is based on null hypothesis test that there is no statistically significant relationship between fitness pleiotropy and gene expression variation after controlling PPI degree, CC.

|  | ρ | p value |
| --- | --- | --- |
| ρ fitness pleiotropy, expression variation | PPI degree | -0.148 | < 2.2e-16 |
| ρ fitness pleiotropy, expression variation | CC | -0.155 | < 2.2e-16 |

Supplemental Table 25. Partial Spearman’s correlation between fitness pleiotropy and PPI degree, CC or CRE. Partial Spearman’s correlation between fitness pleiotropy and PPI degree means Spearman’s correlation after controlling CC and CRE. ρ is Spearman’s correlation coefficient and p-value is based on null hypothesis test that there is no statistically significant relationship between fitness pleiotropy and each measurement after controlling another two measurements.

|  | ρ | p-value |
| --- | --- | --- |
| ρ fitness pleiotropy, CRE | PPI,CC | -0.168 | < 2.2e-16 |
| ρ fitness pleiotropy, PPI | CRE,CC | 0.251 | < 2.2e-16 |
| ρ fitness pleiotropy, CC | CRE,PPI | -0.005 | 0.775 |
